# Characterization of Bamboo Culm as Potential Fibre for Composite Development

**DOI:** 10.3390/ma16145196

**Published:** 2023-07-24

**Authors:** Balkeshwar Singh, Yalew Dessalegn, Melesse Workneh Wakjira, Cherinet Girma, Ali A. Rajhi, Alaauldeen A. Duhduh

**Affiliations:** 1 Department of Mechanical Engineering, Program of Manufacturing Engineering, Adama Science & Technology University, Adama P.O. Box 1888, Ethiopia; balkeshwar71@rediffmail.com (B.S.); melewine@yahoo.com (M.W.W.); 2 Department of Mechanical Engineering, Kombolcha Institute of Technology, Wollo University, Kombolcha P.O. Box 208, Ethiopia; 3 Department of Mechanical Engineering, Program of Automotive Engineering, Adama Science and Technology University, Adama P.O. Box 1888, Ethiopia; cheregir15@gmail.com; 4 Department of Mechanical Engineering, College of Engineering, King Khalid University, Abha 61421, Saudi Arabia; arajhi@kku.edu.sa; 5 Department of Mechanical Engineering Technology, CAIT, Jazan University, Prince Mohammed Street, Jazan 45142, Saudi Arabia; adahduh@jazanu.edu.sa

**Keywords:** bamboo age, bamboo species, culm height, harvesting season, physical properties

## Abstract

This study aims to evaluate how age, harvesting seasons, and culm height affect the properties of various bamboo species. The properties of bamboo fibres for composite development in Ethiopia have not been investigated so far. In this study, the properties of *Y. alpina* and *B. oldhamii* were scientifically investigated for bamboo culm structural applications and bamboo fibre composite development based on age and the harvesting season. *Y. alpina* was collected at Injibara and Mekaneselam which are located in east Gojjam and south wollo, whereas *B. oldhamii* was collected at Kombolcha which is located in south Wollo, Ethiopia. Three representatives of bamboo plants were collected in the three regions, namely from three age groups, across two harvesting months. The highest and lowest moisture content and shrinkage were measured at the ages of one year and three years, respectively, whereas basic densities were measured at the ages of three years and one year. The harvest month of November yields higher moisture content and shrinkage but lower basic densities compared to February. *Yushania alpina* has a higher moisture content and shrinkage but lower basic densities compared to *Bamusa oldhamii*. The current research demonstrates that the three-year-old groups and the harvesting month of February produce yields more suited for construction and structural purposes due to the ensuing good dimensional stability after drying. From the highest to the lowest percentage of the degree of crystallinity of the yield, it is that derived from Inj., followed by Meka., and then Kombolcha, respectively. Bamboo fibres have high powder crystals and degradation temperatures which make them suitable for composite development at two year old. *Yushania alpina* has a higher degree of crystallinity and degradation temperature of cellulose compared to *Bambusa oldhamii*.

## 1. Introduction

One of Africa’s most prosperous bamboo resource bases is in Ethiopia. However, the ability of bamboo forests to bring further socioeconomic and environmental advantages has been constrained due to a lack of accurate data on bamboo resources. Construction, traditional furniture, food, musical instruments, and medicine are Ethiopia’s top five applications of bamboo. Food, medicine, and musical instruments are among the socioeconomic applications of bamboo that are statistically significant [1]. The demand for construction materials rises in tandem with rising populations and aspirations. A lack of industry and infrastructure makes it difficult for developing nations in Asia and Africa to satisfy this demand [2]. Countries like Indonesia, Thailand, Vietnam, and Myanmar must rely on imported building supplies like sand, cement, steel, and wood to meet the rising demand for housing and infrastructure in new cities [3]. However, this can be an issue in the long run because these building materials are either difficult to come by locally (like sand or wood) or can only be imported (copper, iron ore, steel, or other metals). If alternatives are not identified, many nations may not be able to satisfy the growing demand for construction materials. Undoubtedly, plans are rising to replace wood and steel with locally accessible renewable, affordable, and sustainable building materials [4].

There are many different types of bamboo; they may grow in tropical, subtropical, and mild temperate climates. The most recent Global List of Bamboos, which updated information from the Global List of Bamboos and Rattans in 2016, ref. [5] states that there are 130 genera and 1700 species of bamboo worldwide [6]. Only a small number of bamboo species have been successfully planted and harvested. For instance, China’s bamboo board manufacturing industry virtually exclusively uses Moso bamboo as a raw material (*Phyllostachys edulis*). Many high-quality bamboo resources still have not been adequately developed or used. Some species of clumped bamboo, which are common in southeast Asia and south Asia are notably neglected. Since clumped bamboo lacks a robust rhizome system, it will not significantly expand its growing area, making it simpler to control than dispersed bamboo. As a result, this variety of bamboo has a particular potential for development [7].

According to remote sensing data, China has 6.5 million hectares of bamboo forest, with 40 genera and 800 species [8]. Similar research in east African nations yielded the following estimates of resources of bamboo: Ethiopia has 1,438,705 ha of highland and lowland bamboo resources covered; Kenya has 131,040 ha of resource of bamboo covered; and Uganda has 54,587 ha of resource of bamboo covered [9]. Tanzania and Madagascar have a resource of bamboo covering 127,000 ha and 1,123,694 ha, respectively [10].

In comparison to synthetic fibres, natural fibres are regarded as more environmentally benign and biodegradable. The specific tensile strength of bamboo is comparable to that of synthetic fibres. Parameters generated from the physical characteristics of the original bamboo have an impact on the tensile strength of bamboo fibres. The variables that have an impact are the species, age, and bamboo culm positions. The age of the bamboo has a significant impact [11]. According to seasonal research on one species, harvesting in the fall or winter produced larger yields of fibre extraction with better mechanical qualities [10]. The moisture content of bamboo was discovered to decrease as it matures which has a corresponding effect on the strength properties. Bamboo’s properties, such as bending and compressive strength, improve with age. The compressive strength was not affected by the height of the bamboo culm but it was affected by age [12].

Taking into account all the factors, particularly the high mechanical strength and water resistance of bamboo scrimbers, as well as the lower moisture content of mature green bamboo, the four-year-old bamboo culms were preferred for use as raw materials in the production of bamboo scrimbers [13]. The property variation with age and location within a culm was highlighted: Ma bamboo fibres (*Dendrocalamus latiflorus Munro*) that are four years old are stiffer and stronger than fibres that are one year old but the location of the culm is insignificant on the strength. When compared to softwood fibres, Ma bamboo fibres have superior stiffness and strength data. Four-year-old Ma bamboo fibres are stiffer and stronger than one-year-old fibres [14].

Bamboo flattening technology could raise the rate of utilization of bamboo resources from 30–55%, decrease the amount of adhesive used by 30%, and improve the added value by more than 20% [15]. However, due to the hollow cylinder and large curvature of bamboo culms, obtaining crack-free bamboo boards with an ideal width remains a huge challenge. The increased density and mechanical properties of cell walls improved the modulus of elasticity of the bamboo board. Conventional softening methods involving chemical agents and oil are time-consuming and costly. However, softening methods of high-pressure and saturated steam methods of cell wall bamboo are more environmentally friendly, cost-effective, and time-efficient [16].

Bamboo fibre porous materials have the potential to be low-cost and lightweight structural materials. The fibres are utilized to make two kinds of solids: fibrous materials (with external fibrillation and natural binding) and rigid close-celled foams. The mechanical characteristics and obtained density of fibrous networks are linked to the fibre length and the amount of external fibrillation [17].

The purpose of the current study is to describe the characteristics of bamboo species grown in Ethiopia and the impact that age, the harvesting season, and height have on their use in bamboo culm structural applications and fibres’ composite development. Research on the properties of Ethiopian bamboo species as potential fibres for composite development has not been conducted so far. Nowadays, natural fibres substituted with synthetic fibres like carbon and glass fibres are heavy, expensive, not eco-friendly, non-biodegradable, and require a high utilization of energy during processing. Natural fibres did not have data in the manufacturing catalogue that exactly represented their identities. The properties of bamboo fibres were determined by their age, extraction method, harvesting season, method of composite manufacturing, and orientation of the fibres.

## 2. Materials and Methods

### 2.1. Materials

**Figure 1:**
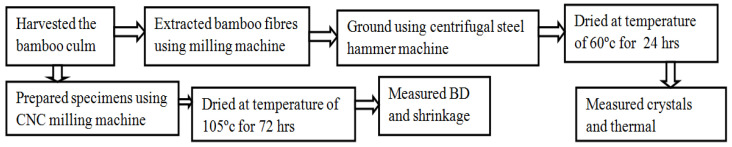
Flow chart of research activities.

As shown in Figure 1, three bamboo plants were harvested in the Inj. (Injibara), Kocha. (Kombolcha), and Meka. (Mekaneselam) regions in November and February of 2021 at 1, 2, and 3 years. Bamboo plants were cut using a sword and axle at the bottom and above 3 cm inter-nodal lengths. Vernier callipers were used to measure the width, thickness, and length of the specimen that was prepared for testing. The bamboo fibres were extracted using a roll-milling technique that was established in the author’s workshop. The bamboo fibres were ground using a centrifugal steel hammer machine. The specimens for measuring shrinkage and BD were prepared using a CNC milling machine.

### 2.2. Location and Sample Collection

The testing sites of the Inj., Kocha., and Meka. are demonstrated in Table 1. From the highest to the lowest recording, altitude was measured in Meka., Inj., and then Kocha.; whereas the maximum temperatures were measured in Kocha., Inj., and Meka. and the maximum annual rainfalls (An.RF) were registered in Inj., Meka., and Kocha., respectively [18,19,20].

### 2.3. Determination of Moisture Content (MC)

Samples used to investigate bamboo’s MC were prepared during harvesting to prevent moisture loss at different areas, ages, harvesting seasons, and culm heights. In total, 18 samples were measured at the ages of 1, 2, and 3 years old, and 6 samples were prepared at each culm height. In total, 54 samples were taken to evaluate the MC in each of the regions of Inj., Kocha., and Meka. The samples were weighed before and after drying using a digital balance with a 0.00001 g precision and they were dried in an oven at 105 ± 2 °C for 72 h until a constant weight was attained. Based on ASTM 2004 criteria, the percentage of MC was evaluated using Equation (1) [21].

(1) $$MCs\left(\%\right)=\frac{M1\left(g\right)-M2(g)}{M2(g)}\times 100\%$$

where MC is the moisture content, M1 is the weight before drying, and M2 is the weight after drying.

### 2.4. Measuring the Basic Density (BD)

The same experiments were conducted as in Section 2.3 to measure the MC. The samples were prepared using a CNC milling machine in the size of 20 mm × 20 mm × thickness of the culm at the time of harvesting. The weight and dimensions of the samples were measured using a digital balance accuracy of 0.00001 gm and a digital micrometer, respectively. The samples were dried for 72 h at 105 ± 2 °C in an oven to achieve a consistent weight. The BDs of the samples were prepared and measured under the ASTM D2395-14 standard test method using the oven-dried weight and the dimension of green volume [ASTM D2395] [22]. BD was measured by using Equation (2).

(2) $$BD(Kg/m^{3})=\frac{ODW(Kg)}{GV(m^{3})}$$

where BD is basic density, ODW is the oven dry weight, and GV is the Green volume.

### 2.5. Bamboo Culm Shrinkage

The same number of experiments were conducted as in Section 2.3 to measure the MC. Similar sample preparations were followed in Section 2.4 to the measure the BD. The bamboo culm shrinkage was prepared and measured under the ASTM 1997 standard test method using the oven-dried weight and green dimension [23]. Bamboo culm shrinkage was evaluated by using Equation (3).

(3) $$SHCM\left(\%\right)=\frac{GD\left(mm\right)-ODD\left(mm\right)}{GD\left(mm\right)}\times 100$$

where SHCM is the bamboo culm shrinkage, GD is the dimension of green, and ODD is the dimension of that oven dried.

### 2.6. Measuring the Degree of Crystallinity (DOC)

The value of the degree of crystallinity indicates that it improves the properties of bamboo fibres when they are used as reinforcement with resin materials. As a result of their degree of crystallinity, bamboo fibres are used for composite development as reinforcement materials in the composite industry.

Scissors were used to trim the fibres of less than 2 mm in length so that they could be hammer milled into a powder with a particle size smaller than 250 μm. The reflection (R) measurement was performed using a Bruker D8 Advance goniometer (Bragg–Brentano geometry) and a copper Kα (λ = 0.154189 nm) X-ray source operating at 40 mA and 40 kV in line focus. Data was gathered across an angular range of 5–60° 2θ using a Lync Eye-XE-T detector with steps of 0.04° 2θ and 0.1 s each step. Using the combined regions under the identified crystalline and amorphous components, the degree of crystallinity was determined. The empirical approach suggested by Segal et al. was utilized to calculate the crystallinity using Equation (4) [20]:

(4) $$DOC\left(\%\right)=\frac{I_{002}-I_{am}}{I_{am}}\times 100\%$$

where DOC is the sample degree of crystallinity; I_002_ is the maximum intensity of (002) the lattice diffraction at 2θ degrees which represents both crystalline and amorphous materials; and I_am_ is the intensity of diffraction at 2θ degrees, representing only amorphous material.

### 2.7. Measuring Thermal Properties of Fibres

To assess a material’s thermal and oxidative stability at temperatures up to 500 °C, TGA specifies the weight loss or increase brought on by decomposition, oxidation, or dehydration. To determine the areas on the weight loss curve with the highest rate of change, the first derivative of the TGA curve (DTG) is typically calculated as well. TGA for technical fibres was carried out at room temperature on an SDT Q600 T.A. instrument. The experiment was carried out at a flow rate of 20 mL/min and a heating rate of 5 °C/min in an inert atmosphere. The TGA measurements were performed on samples ranging in size from 5 to 12 mg. Thermal analysis makes use of TGA/DTG curves for fibres in argon environments.

### 2.8. Measuring Single Fibres: The Tensile Test

Before tensile testing, the fibres were visually selected to ensure that there were no defects along their length. To ensure a good grip and straight position between the clamps, both ends of the fibre samples were glued with cyanoacrylate adhesive onto a paper frame that was cut after being clamped by the testing machine’s jaws. The abrasive paper frame allows for easier sample mounting and fibre alignment in the grips, while the abrasive paper reduces fibre slippage during the test. The tensile strength of single fibre test is measured based on ASTM C1557 standard on an Instron 5943 equipped with a 100 N load cell at a crosshead displacement of 1.5 mm/min and a conditioned environment of 50% RH and 21 °C [24].

### 2.9. Statistical Analysis

The physical properties of bamboo species in Ethiopia were determined using ANOVA Stata Version 12 software to identify the significant difference in the mean of the results. The acceptable significance level is a *p* < 0.05 value. The results of the experiments evaluated the factors which are either significant or insignificant in the properties of bamboo.

## 3. Result and Discussion

### 3.1. Measurement of MC

**Figure 2:**
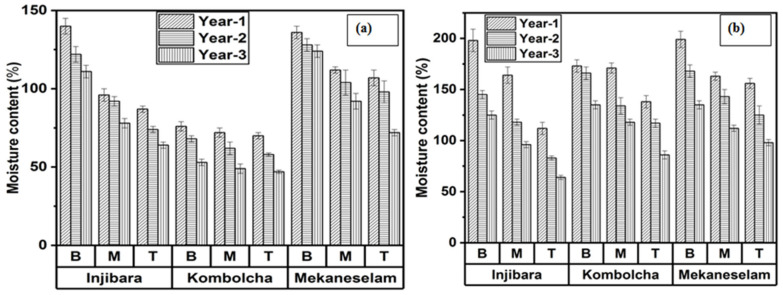
Ethiopian bamboo species MC in (**a**) February and (**b**) November.

Figure 2a,b depicts the effects of the age, culm height, and bamboo species. According to the current research findings, the MC decreases when age increases and moves along the height from bottom (B) to top (T). The middle parts of the bamboo culm are represented by M. As shown in Figure 2a, one-year-old bamboo of Inj., Kocha., and Meka. are measured with the highest MC of 107%, 72%, and 118%, whereas three-year-old bamboo measured the lowest MC of are 84%, 49%, and 96%, respectively. Moreover, the bottom parts of Inj., Kocha., and Meka. bamboo measured the highest MC, which were 124%, 66%, and 129%, whereas the top parts measured the lowest MC, which were 75%, 58%, and 92%, respectively. The MC was higher than 100%, indicating that the bamboo culm did not develop more fibre sheaths at an early stage rather than a greater amount of parenchyma. The harvesting month affects the MCs which showed the lowest MC in February compared to November; so, the harvesting month of February of bamboo culm was used for the applications of construction and structure. In Figure 2b, one-year-old bamboo of Inj., Kocha., and Meka. measured the highest MCs which were 158%, 160%, and 172%, whereas 3-year-old bamboo measured the lowest MCs of 95%, 113%, and 115%, respectively. Moreover, the bottom portion of Inj., Kocha., and Meka. measured the highest MCs, which were 156%, 158%, and 167%, whereas the top parts measured the lowest MCs, which were 86%, 113%, and 126%, respectively. The highest to the lowest MC of Ethiopian bamboo species in February were Meka., Inj., and Kocha., whereas in November, they were Meka., Kocha., and Inj., respectively. November was the best month for fibre extraction for composite development due to the higher amount of water that exists in the culm. A younger age did not yield much development of fibre bundles in the culm so more moisture content exists in the culm, whereas the matured bamboo developed more fibre bundles in the culm so it has a low moisture content.

Along with height, the MC of the bamboo *species Bambusa balcooa, Bambusa tulda, Bambusa salarkhanii,* and *Melocanna baccifera* decreased. In contrast to earlier research on bamboo species, Ethiopian bamboo species have higher MC in February [25]. *Yushanina alpina bamboo,* which is two and three years old, has MC of 120% and 100%, respectively, which are greater values compared to the present findings of bamboo species in Ethiopia in February [10]. One, two, and three-year-old *Bambusa vulgaris* var. *vulgaris* has MCs of 49.13%, 40.07%, and 39.73%, whereas *Bambusa vulgaris* var. *striata* has MCs of 29.68%, 29.43%, and 27.82%, *Bambusa balcooa* has MCs of 56.86%, 51.46%, and 48.35%, *Bambusa tulda* has MCs of 42.93%, 48.76%, and 38.60%, *Bambusa polymorpha* has 68.14%, 62.48%, and 56.85%, *Dendrocalamus strictus* has 49.02%, 48.76%, and 38.60%, and *Bambusa bambusa* has 56.19%, 49.64%, and 41.34%, respectively. They had lower MCs than Ethiopian bamboo species at one, two, and three years old in February [26].

The MC at the age of one, two, and three years old of *Bambusa Blumeana* are (79.3–97.0%) lower, (57.3–79.6%) lower, and (57.5–79.6%) higher, whereas *Bambusa blumeana* is (105.92%) lower, (81.33%) lower, and (94.80%) higher, *Bambusa Vulgaris var* is (224.96%) higher, (86.66%) lower, and (89.95%) higher, and *Gigantochlo scortechinii* is (108.58%) lower, (93.21%) lower, and (90.57%) higher compared to the current finding of Ethiopian bamboo species at the age of one, two, and three years old in November, respectively [27,28].

### 3.2. Measurement of BDs

**Figure 3:**
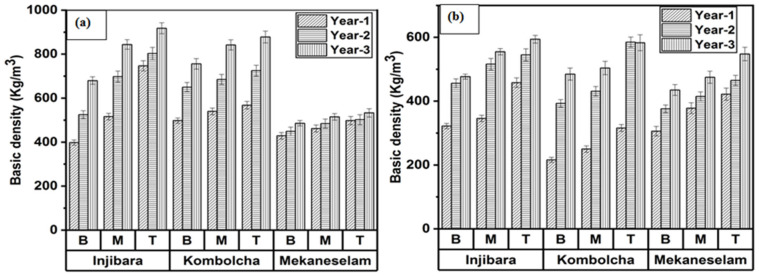
Ethiopian bamboo species’ BDs are (**a**) February and (**b**) November.

Figure 3a,b depicts the effect of the age, height of bamboo culm, and species of bamboo culm on BD. In the current findings, the BDs increase as the culm age increases and move along the height from B to T. As shown in Figure 3a, that of three years old in Inj., Kocha., and Meka. measured the highest BDs of 814 Kg/m^3^, 524 Kg/m^3^, 486 Kg/m^3^, whereas that which was one year old measured the lowest BDs of 553 Kg/m^3^, 260 Kg/m^3^, and 369 Kg/m^3^, respectively. Moreover, the bottom portion of Inj., Kocha., and Meka. measured the lowest BDs, which were 534 Kg/m^3^, 364 Kg/m^3^, and 372 Kg/m^3^, whereas the top portions measured the highest BDs, which were 822 Kg/m^3^, 494 Kg/m^3^, and 478 Kg/m^3^, respectively. In Figure 3b, that of three years old in Inj., Kocha., and Meka. measured the highest BDs which were 542 Kg/m^3^, 825 Kg/m^3^, and 511 Kg/m^3^, whereas that of one year old measured the lowest BD of 375 Kg/m^3^, 535 Kg/m^3^, and 463 Kg/m^3^, respectively. Moreover, the bottom portion of Inj., Kocha., and Meka. measured the lowest BDs, which were 418 Kg/m^3^, 634 Kg/m^3^, and 454 Kg/m^3^, whereas the top portions measured the highest BDs, which were 532 Kg/m^3^, 723 Kg/m^3^, and 511 Kg/m^3^, respectively.

The highest to the lowest BDs of Ethiopian bamboo species in February were Inj., Kocha., and Meka., whereas in November, Kocha., Inj., and Meka. were the highest to the lowest, respectively. Kocha. and Inj. have good bamboo culm properties for construction and structural application in the harvesting month of November and February, respectively. Matured bamboo measured as having higher BDs compared to younger due to more fibre bundle development in the culm, whereas the harvesting month of February measured higher BDs compared to November due to the lower moisture content in February.

As shown in Table 2, BDs at the age of two and three years old of *Bambusa vulgaris* var. *vulgaris*, *Bambusa balcooa*, *Bambusa tulda*, *Bambusa polymorpha*, *Dendrocalamus strictus*, and *Bambusa bambos* were lower compared to the current findings of Ethiopian bamboo species but they had higher BDs compared to Ethiopian bamboo species at the age of one year old. *Bambusa vulgaris* var. *striata* had a lower BD compared to the current findings of Ethiopian bamboo species at the ages of one, two, and three years old in February [26]. Comparing current findings of Ethiopian bamboo species at the ages of one, two, and three years old in February, the BDs of *Bambusa-balcooa* and *Bambusa-tulda* were lower at between two and four years old. In contrast, *melocanna-baccifera* and *Bambusa-salarkhanii* had higher BDs [25]. *Yushania alpina* and *Bamusa vulgaris* var. had higher BDs at the age of one, two, and three years old, whereas *Bamusa vulgaris* var. and *Gigantochlo scortechinii* had lower values compared to the current findings of Ethiopian bamboo species at the age of one, two, and three years old in November [10,27]. The BD of *Gigantochloa* at two and four years old is two-fold (733 Kg/m^3^) higher and four-fold (751 Kg/m^3^) higher, respectively, while the BDs of Ethiopian bamboo species in November were lower along the culm height at the bottom (697 Kg/m^3^), middle (751 Kg/m^3^), and top (759 Kg/m^3^) [29].

### 3.3. Measurement of Radial Shrinkage (RASH)

**Figure 4:**
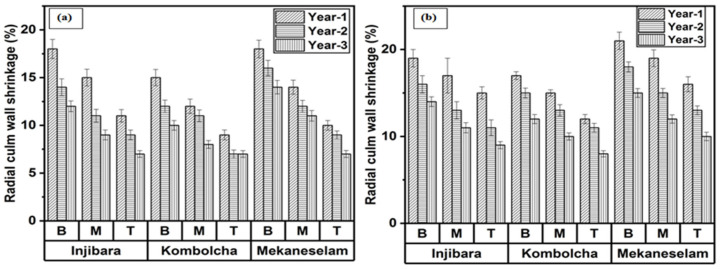
Ethiopian bamboo species’ RASH in (**a**) February and (**b**) November.

Figure 4a,b depicts the effect of age, bamboo culm height, and bamboo culm species on radial shrinkage. RASH decreases when the age increase and moves a long height from B to T. As shown in Figure 4a, one year olds of Inj., Kocha., and Meka. measured the highest percentage of RASH which were 14%, 12%, and 14%, whereas three-year-old specimens measured the lowest percentage of RASH, which were 9%, 8%, and 10%, respectively. Moreover, the bottom portion of Inj., Kocha., and Meka. measured the highest RASH, whereas the top portions measured the lowest percentage of RASH, which were 15%, 12%, and 16%, respectively. In Figure 4b, the one year old of Inj., Kocha., and Meka. measured the highest percentage of RASH which were 17%, 14%, and 18%, whereas three years old measured the lowest percentage of RASH, which were 11%, 10%, and 12%, respectively. Moreover, the bottom portion of Inj., Kocha., and Meka. measured the highest percentage of RASH, which were 16%, 14%, and 18%, whereas the top portions measured the lowest percentage of RASH, which were 11%, 10%, and 13%, respectively.

The RASH of *Bambusa Blumeana* decreased with age and from the bottom to the top along from the culm height. The current research findings on Ethiopian bamboo species in February showed a lower percentage of RASH than *Bambusa Blumeana* [28]. The RASH of bamboo species in Ethiopia in February was lower percentage of than that of *Gigantochlo Scortechinii* [30]. The current research findings of Ethiopian bamboo species at the ages of one to three years old in November showed a higher percentage of RASH than that of *Gigantochloa levis* at the age of two to four years old [29]. The percentage of RASH decreased along the culm height of *B. balcooa*, *B. tulda*, *B. salarkhanii*, and *Melocanna baccifera* from bottom to top. The shrinkage in wall thickness has not been significantly affected by bamboo culms older than four years. Similar results were noted with the most recent findings of Ethiopian bamboo species [25].

### 3.4. Measurement of Tangential Shrinkage (TASH)

**Figure 5:**
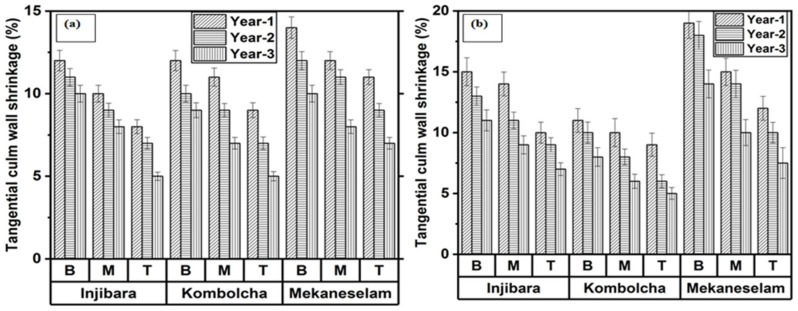
Ethiopian bamboo species’ TASH in (**a**) February and (**b**) November.

Figure 5a,b depicts the effect of age, bamboo culm height, and bamboo culm species on tangential shrinkage. TASH decreases when the age increases and moves along the height from B to T. As shown in Figure 5a, one-year-old specimens of Inj., Kocha., and Meka. measured the highest percentage of TASH of 10%, 11%, and 12%, whereas three years old measured the lowest percentage of TASH of 7%, 7%, and 8%, respectively. Moreover, the bottom portion of Inj., Kocha., and Meka. measured the highest TASH, which were 11%, 10%, and 10%, whereas the top portions measured the lowest percentage of TASH, which were 7%, 7%, and 9%, respectively. In Figure 5b, oneyearold specimens of Inj., Kocha., and Meka. measured the highest TASH of 13%, 10%, and 15%, whereas three-year-old specimens measured the lowest percentage of TASH of 9%, 6%, and 10%, respectively. Moreover, the bottom portion of Inj., Kocha., and Meka. measured the highest percentage of TASH, which were 13%, 9%, and 14%, whereas the top portions measured the lowest percentage of TASH, which were 8%, 6%, and 9%, respectively.

### 3.5. Physical Properties Using ANOVA

#### 3.5.1. Moisture Content

Table 3 shows the impact of age, culm height (CH), and harvested month (HM) on the MC of various bamboo species in Ethiopia. The static significance value of the *p*-value at the factors of age, CH, and HM on the MC is less than 0.05 but the harvest month of Kocha. is not a significant value at *p* < 0.05 on the MC. The statistical value indicates that age, CH, and HM have an impact on the value of the MC on the bamboo culm which is either for construction and structural applications or fibres extraction. The amount of moisture content has a positive impact on the time of fibre extraction but it harms the utilization of the structural application.

#### 3.5.2. Basic Density

Table 4 shows the impact of age, CH, and HM on the MC of various bamboo species in Ethiopia. The BD of Inj., Kocha., and Meka. are statistically determined based on age, CH, and HM using ANOVA. The age, CH, and HM of Inj. bamboo culm influenced the BD and an effect was observed at a significant value of *p* < 0.05, whereas the age and CH of Kocha. bamboo culm influenced the BD and an effect was observed at a significant value of *p* < 0.05. However, HM did not influence the BD and an effect was not observed at an insignificant value of *p* < 0.05. The age and HM of Meka. bamboo culm influenced the BD and an effect was observed at a significant value of *p* < 0.05 but CH did not influence the BD and an effect was not observed at an insignificant value of *p* < 0.05 on the BD of the bamboo culm. The value of BD determined the construction and structural applicability of the bamboo culm. The age and harvested month of the bamboo culm influenced the BDs which determined the area construction and structural applications.

#### 3.5.3. Radial Shrinkage

The RASH of Inj., Kocha., and Meka. are presented in Table 5. The age, CM, and HM of Inj. and Meka. (*Y. alpina*) bamboo culm is a significant value at *p* < 0.05 on the RASH of the bamboo culm, whereas the age and CH of Kocha. (*B. oldhamii*) bamboo culm is a significant value at *p* < 0.05 but HM is an insignificant value at *p* < 0.05 on the RASH of the bamboo culm. The percentage of RASH determined the dimensional stability of the bamboo culm which is utilized in construction and structural applications.

#### 3.5.4. Tangential Shrinkage

The effect of age, CH, and HM on the TASH is presented in Table 6. The age, CH, and HM of Inj., Kocha., and Meka. are significant values at *p* < 0.05 on the TASH of the bamboo culm. The bamboo culm cracked and ruptured during the drying season due to the associated unproportional and higher TASH. The construction and structural application of the bamboo culm is determined based on the TASH of the bamboo species.

### 3.6. Bamboo Fibres Thermal Properties

The thermal gravimetric analyses (TGA) and derivatives (DTG) curves for Ethiopian bamboo fibres under argon atmospheres based on ages are demonstrated using figures. At temperatures below 105 °C, all samples exhibit a steady loss of mass that is linked to the release of water. Only one identifiable peak in the DTG curves was validated by the decomposition of the lignocellulose components (hemicelluloses, cellulose, and lignin) for all bamboo fibres.

#### 3.6.1. Injibara Bamboo Fibres

**Figure 6:**
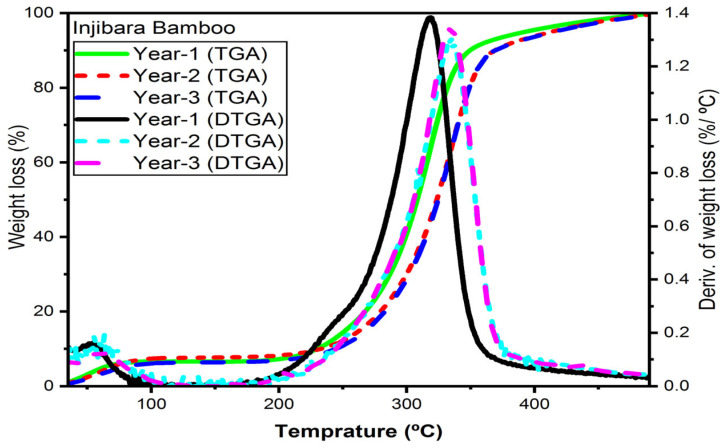
TGA/DTGA of Injibara bamboo fibres in inert argon gas.

The TGA/DTGA of Inj. bamboo fibres based on age are presented in Figure 6. The moisture of the bamboo powder is released below 100 °C. At ages one, two, and three years, cellulose’s thermal decomposition temperatures are 319 °C, 328 °C, and 328 °C, respectively, whereas hemicelluloses’ temperatures are 260 °C, 291 °C, and 284 °C and lignin’s temperatures are 390 °C, 410 °C, and 410 °C. The thermal degradation of fibres was determined by their ages of maturity. The degradation temperature of younger bamboo fibres was lower than that of mature ones. The degradation temperature was constant when the lignification of the bamboo fibres was completed. In comparison to the *Bamboosa vulgaris* fibres in their natural form (220 °C), a rise in the early degradation temperature (291.0 and 284.0 °C) at the ages of two and three years old, respectively, can be noticed. The initial degradation temperature of *Bamboosa vulgaris* was found to have low thermal behaviour [29].

The current research findings show that the initial degradation temperature of hemicelluloses at the ages of one, two, and three years is 5%, 20%, and 14% higher than bamboo pulp fibres [31]; 4%, 18%, and 12% higher than *Bambusoideae* bamboo [32]; 15%, 28%, and 23% higher than Bamboosa Vulgaris [33]; and 9%, 23%, and 17% higher than bamboo fibres [34], respectively.

The initial degradation temperature of cellulose at the ages of one, two, and three years is 11%, 9%, and 10% lower than bamboo pulp fibres [31]; 16%, 14%, and 15% lower than *Bambusoideae bamboo* [32]; and 14%, 11%, and 13% lower than bamboo fibres [34]; however, they are 6%, 9%, and 8% higher than *Bamboosa vulgaris* [33], respectively.

#### 3.6.2. Kombolcha Bamboo Fibres

**Figure 7:**
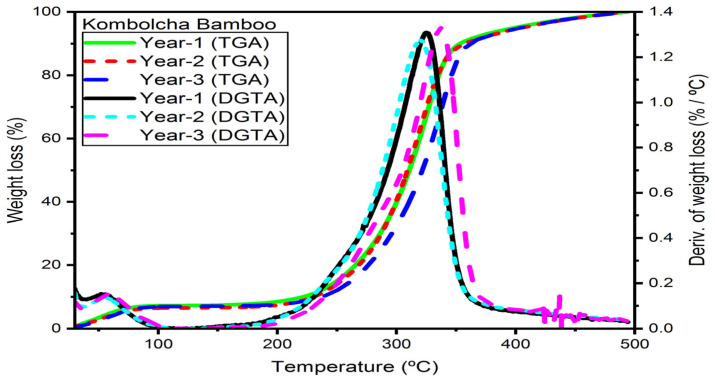
TGA/DTG of Kombolcha bamboo fibres in an environment of argon.

The TGA/DTGA of Kocha. bamboo fibres based on age are presented in Figure 7. The thermal decomposition temperatures at the ages of one, two, and three years old of cellulose is 326 °C, 322 °C, and 332 °C, whereas hemicelluloses are 273 °C, 273 °C, and 285 °C and lignin is 415 °C, 425 °C, and 425 °C, respectively.

The age of two years has a lower percentage of weight loss compared to ages one and three. The ages of two and three years old are also noticed and early-stage degradation is defined by a rise in the early degradation temperatures, which has increased with the thermal stability of the fibre.

The early degradation temperature of hemicelluloses at the ages of one, two, and three years is 10%, 13%, and 7% higher than bamboo pulp fibres [31]; 8%, 12%, and 6% higher than *Bambusoideae bamboo*; 19%, 23%, and 17% higher than *Bamboosa vulgaris* [33]; and 14%, 17%, and 15% higher than bamboo fibres [34], respectively. The current research findings demonstrate that the initial degradation temperature of cellulose at the ages of one, two, and three years is 9%, 10%, and 6% lower than bamboo pulp fibres [31]; 14%, 15%, and 11% lower than *Bambusoideae bamboo* [32]; and 11%, 12%, and 8% lower than bamboo fibres [34]; however, it is 8%, 7%, and 11% higher than *Bamboosa vulgaris* [33], respectively.

#### 3.6.3. Mekaneselam Bamboo Fibres

**Figure 8:**
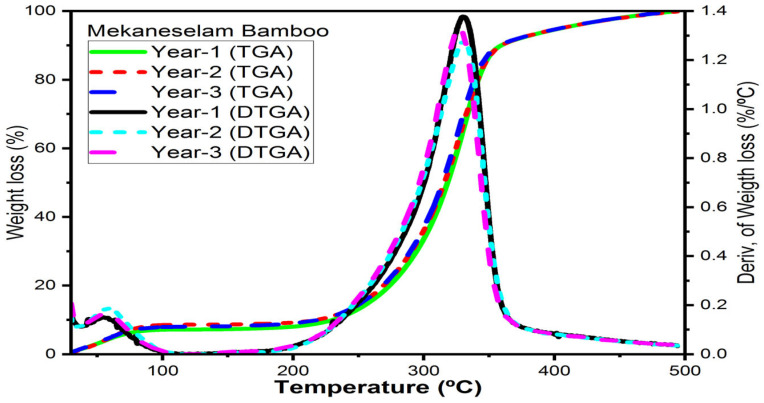
TGA/DTG of Mekaneselam bamboo fibres in the environment of argon.

Figure 8 illustrates how age affects the thermal decomposition of Meka. bamboo fibres. At the ages of one, two, and three years, cellulose undergoes thermal breakdown at 329 °C, 330 °C, and 329 °C, respectively. The decomposition temperature of hemicelluloses and lignin is 281 °C and 424 °C, respectively. All ages of Meka. bamboo fibres have a similar decomposition temperature as hemicelluloses as well as lignin content, whereas one-year-old Meka. bamboo has a higher percentage of weight loss compared to those of two and three years. The decomposition temperature of cellulose is higher and suitable for polymer composite development.

The current research findings demonstrate that the initial degradation temperature of hemicelluloses at the ages of one, two, and three years is 12%, 13%, and 11% higher than bamboo pulp fibres [31]; 11%, 12%, and 10% higher than *Bambusoideae bamboo* [32]; 22%, 23%, and 21% higher than *Bamboosa vulgaris* [33]; and 16%, 17%, and 15% higher than bamboo fibres [34], respectively. The initial degradation temperature of cellulose at the ages of 1, 2, and 3 years is 9%, 8%, and 9% lower than bamboo pulp fibres, 13%, 14%, and 13% lower than *Bambusoideae bamboo* [32]; and 11%, 10%, and 11% lower than bamboo fibres [34]; however, it is 9%, 10%, and 9% higher than *Bamboosa vulgaris* [33], respectively.

The current research results indicate that the decomposition temperature of bamboo fibres is measured using TGA. The current research findings indicate that the thermal properties of cellulose bamboo fibres have higher decomposition temperatures which are used for thermoplastic and epoxy materials for composite development.

### 3.7. Degree of Crystallinity

The XRD convolution technique, which separates the amorphous and crystalline contributions from the diffraction spectrum, can be used to assess the degree of crystallinity of a polymer. Crystallinity can be determined by dividing the sum of the integrated areas beneath the XRD peaks by the integrated areas of all the crystalline peaks.

#### 3.7.1. Injibara Bamboo Fibres

**Figure 9:**
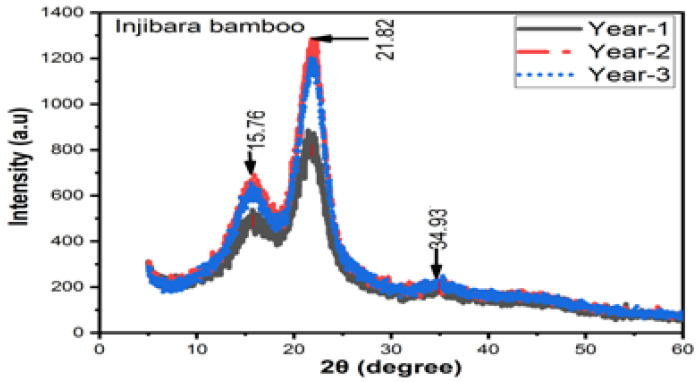
XRD results of Injibara bamboo fibres.

Figure 9 illustrates how aging affects the DOC of Inj. bamboo fibres. At one, two, and three years old, the DOC is 69%, 73%, and 72%, respectively. In comparison to one and three years, bamboo fibres that are two years old have a higher intensity and DOC, whilst bamboo fibres that are one year old have the lowest intensity and DOC. Inj. bamboo fibres have the highest intensity and more powder crystals at 2θ of 21.82° so it is the best fibre type for composite development. The DOC and amorphousness at 2θ are 21.82° and 15.76°, respectively, which has more powder crystals at the age of two years old but lower amounts of powder crystals are found at the age of one years old. However, some silicon content is present at 2θ of 34.93°. The DOC at the ages of one, two, and three years old is 20%, 25%, and 24% higher than *Neosinocalamus affinis* of (55%) [35], and 22%, 26%, and 25% higher than *Moso bamboo* fibres (53.7%) [36]; moreover, it is 7%, 12%, and 10% higher than bamboo pulp fibres (64.3%), respectively [31].

#### 3.7.2. Kombolcha Bamboo Fibres

**Figure 10:**
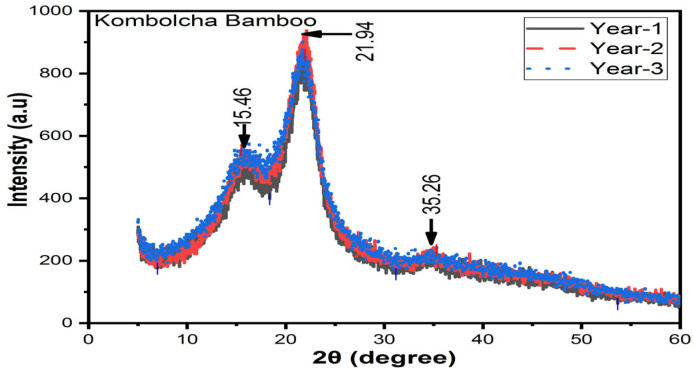
XRD results of Kombolcha bamboo fibres.

Figure 10 illustrates how aging affects the DOC of Kocha bamboo fibres. At one, two, and three years old, the DOC is 58%, 66%, and 62%, respectively. Bamboo fibres that are 2 years old have a higher intensity and DOC than fibres that are one and three years old, although one-year-old fibres have the lowest intensity and degree of crystallinity. At 2θ, the amorphous index is 15.46° and the crystallinity index is 21.94°, respectively. However, a small amount of silicon content at 2θ showed 35.26°. The DOC at the ages of one, two, and three years is 5%, 17%, and 11% higher than *Neosinocalamus affinis* of (55%) [35] and 7%, 19%, and 13% higher than *Moso* bamboo fibres (53.7%) [36]; moreover, that of one and three years is 10% and 4% higher than bamboo pulp fibres, respectively. Furthermore, that which is two years old is 3% higher than bamboo pulp fibres. However, that of one and three years old is 10% and 4% lower than bamboo pulp fibres [31].

#### 3.7.3. Mekaneselam Bamboo Fibre

**Figure 11:**
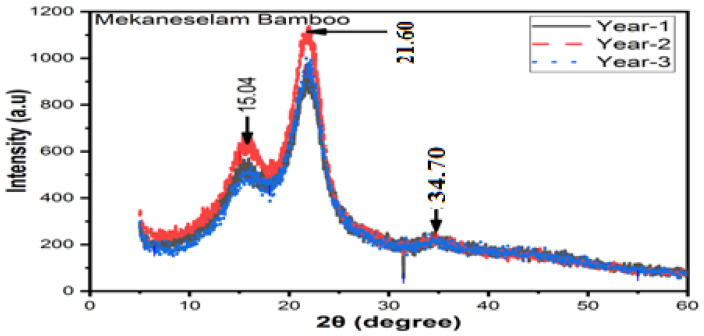
XRD results of Mekaneselam bamboo fibres.

Figure 11 shows the impact of age on the DOC of Meka bamboo fibres. At one, two, and three years old, the DOC is 66%, 68%, and 67%, respectively. Compared to that which is one and three years old, that which is two years old has a higher intensity and DOC but one- and three-year-old fibres have identical intensity and DOC. Fibres which are two years old have a higher order of crystals and more powder crystals so are suitable for composite development. The Meka. bamboo fibres have a higher intensity and more powder crystals at 2θ of 21.60° compared to Kocha. The DOC and amorphous index at 2θ are 21.60° and 15.04°, respectively. However, some silicon content at 2θ has 34.70°. At one, two, and three years old, the DOC is 17%, 19%, and 18% higher than *Neosinocalamus affinis* of (55%) [35] and 19%, 21%, and 20% higher than *Moso* bamboo fibres (53.7%) [36]; moreover, they are 3%, 5%, and 4% higher than bamboo pulp fibres (64.3%), respectively [31].

The authors summarized the current research findings showing that one of the properties of the fibres is determined by the percentage of crystallinity degree and decomposition temperature. The degree of crystallinity of bamboo fibres powder is measured using XRD. The results indicated that bamboo fibres in Ethiopia has a higher degree of crystallinity compared to other natural fibre-reinforced polymer composites. The value of the degree of crystallinity indicates that the properties of bamboo fibres have been improved when used as reinforcement with resin materials.

### 3.8. Measurement of Tensile Strength of Single Bamboo Fibres

The single bamboo fibres’ tensile strength of Ethiopian bamboo species based on the gauge length (GL) is presented in Table 7. The tensile strength and Young’s modulus are decreased when the gauge length is increased. The maximum Young’s modulus (Emax.) is lower than the corrected Young’s modulus (Ecor.) due to slippage of fibres during tensile testing, which then increased the strain to failure. The corrected strain to failure (εcor.) is calculated based on the constant value of the properties of fibres and gauge length. From the highest to the lowest single fibres, the ultimate tensile strength (UTS) and Young’s modulus are Inj., Meka., and Kocha.

## 4. Conclusions

Ethiopian bamboo species’ MC, BDs, RASH, TASH, and DOC, and thermal properties of the fibres are examined concerning their age, harvesting season, and culm height.

- Age, harvesting season, and culm height have a significant effect on the MC, BDs, and culm shrinkages which have influenced the construction, structure, and composite development;
- One-year-old bamboo culm has higher MC and culm shrinkage compared to three-years-old culm due to immature cell development at the early stage, but three-year-old culm has a higher BD and lower MC so it is used for structural and construction applications;
- The harvesting season of November yields a higher MC, which can easily extract bamboo fibres without damage for composite development;
- Two-year-old bamboo fibres have the highest percentage of the degree of crystallinity, which indicates more powder crystal and thus a high strength that resists chemical deterioration.
- Cellulose has a higher decomposition temperature compared to hemicelluloses but it has a lower decomposition temperature compared to lignin;
- Age and bamboo species have a significant effect on the degree of crystallinity and decomposition temperature for composite development;
- Two-year-old bamboo fibres have the highest thermal decomposition temperature of celluloses which indicates more powder crystals and thus that it has a high strength and resists chemical deterioration;
- The current research results finding show that the thermal degradation temperature of the cellulose is above 310 °C which is the highest value for the consolidation temperature of thermoplastics used for polymer composite development;
- Inj. and Meka. bamboo fibres (*Y. alpina*) have a potential for composite development due to their higher degree of crystallinity and thermal degradation temperature compared to Kocha. bamboo fibres (*B. oldhamii*).

## Figures and Tables

**Table 1:** Descriptions of administrative and climatic conditions of the testing sites.

Name of Testing Sites	Testing Regions				Ave. Value of Climate		
	Zone	Region	Lat-Long	Alt. (m)	An.RF (mm)	Maximum Temp. (°C)	Minimum Temp. (°C)
Inj.	Awi	Amahara	10°59′ N 36°55′ E	2540–2865	1813	24	14
Kocha.	S/wollo	Amahara	11°5′ N 39°44′ E	1842–1915	1027	26	20
Meka.	S/wollo	Amahara	10°45′ N 38°45′ E	2605–3000	1048	21	10

**Table 2:** Basic densities of various bamboo species [10,25,26,27,29].

Bamboo Species	Age (Years)	Basic Density (Kg/m^3^)
*Bambusa vulgaris* var. *vulgaris*	1	411
	2	426
	3	442
*Bambusa vulgaris* var. *striata*	1	390
	2	410
	3	435
*Bambusa balcoola*	1	428
	2	442
	3	466
*Bambusa tulda*	1	431
	2	447
	3	468
*Bambusa polymorpha*	1	417
	2	428
	3	442
*Dendro calamus* strictus	1	432
	2	449
	3	458
*Bambusa bambos*	1	422
	2	439
	3	451
*Yushania alpina*	1	600
	2	653
	3	667
*Bambusa blumeana*	1	1103
	2	1037
	3	1000
*Bambusa vulgaris* var.	1	293
	2	507
	3	543
*Gigantochlo scoretechinii*	1	470
	2	533
	3	557

**Table 3:** Statistical analysis of MC.

Factors	Inj.		Kocha.		Meka.	
	*p*-Value	*p* < 0.05	*p*-Value	Sign. (*p* < 0.05)	*p*-Value	*p* < 0.05
Age	0.0000	Yes	0.0000	Yes	0.0000	Yes
CH	0.0000	Yes	0.0000	Yes	0.0000	Yes
HM	0.0002	Yes	0.2738	No	0.0000	Yes

Note: CH is an abbreviation for culm height and HM is an abbreviation for the harvest month.

**Table 4:** Statistical analysis of BD.

Factors	Inj.		Kocha.		Meka.	
	*p*-Value	*p* < 0.05	*p*-Value	*p* < 0.05	*p*-Value	*p* < 0.05
Age	0.0000	Yes	0.0000	Yes	0.0000	Yes
CH	0.0000	Yes	0.0000	Yes	0.1968	No
HM	0.0000	Yes	0.9239	No	0.0000	Yes

**Table 5:** Statistical analysis of RASH.

Factors	Inj.		Kocha.		Meka.	
	*p*-Value	*p* < 0.05	*p*-Value	*p* < 0.05	*p*-Value	*p* < 0.05
Age	0.0000	Yes	0.0000	Yes	0.0000	Yes
CH	0.0000	Yes	0.0000	Yes	0.0000	Yes
HM	0.0029	Yes	0.0286	No	0.0000	Yes

**Table 6:** Statistical analysis of TASH.

Factors	Inj.		Kocha.		Meka.	
	*p*-Value	*p* < 0.05	*p*-Value	*p* < 0.05	*p*-Value	*p* < 0.05
Age	0.0000	Yes	0.0000	Yes	0.0000	Yes
CH	0.0000	Yes	0.0000	Yes	0.0000	Yes
HM	0.0000	Yes	0.0022	Yes	0.0000	Yes

**Table 7:** Measurement of ultimate tensile strength of single bamboo fibres.

Bamboo Species	GL (mm)	Emax. (GPa)	Ecor. (GPa)	UTS (MPa)	εmax. (%)	εcor. (%)
Inj.	15	50 ± 5	52 ± 5	600 ± 80	1.31 ± 0.11	1.24 ± 0.11
	25	43 ± 4	45 ± 3	580 ± 70	1.45 ± 0.13	1.54 ± 0.13
	30	35 ± 4	37 ± 3	553 ± 60	1.69 ± 0.14	1.45 ± 0.12
	40	29 ± 3	31 ± 3	542 ± 55	1.6 ± 0.13	1.53 ± 0.13
	50	26 ± 3	29 ± 3	432 ± 45	2.09 ± 0.18	1.56 ± 0.12
Kocha.	15	41 ± 5	43 ± 5	508 ± 50	1.09 ± 0.09	1.05 ± 0.08
	25	34 ± 3	36 ± 4	470 ± 45	1.18 ± 0.08	1.1 ± 0.06
	30	29 ± 3	31 ± 3	385 ± 40	1.16 ± 0.09	1.12 ± 0.09
	40	27 ± 3	28 ± 3	378 ± 35	1.52 ± 0.13	1.45 ± 0.12
	50	20 ± 2	22 ± 2	370 ± 35	2.18 ± 0.19	1.57 ± 0.13
Meka.	15	46 ± 5	48 ± 6	566 ± 60	1.15 ± 0.11	1.07 ± 0.09
	25	41 ± 4	43 ± 5	528 ± 55	1.43 ± 0.12	1.34 ± 0.11
	30	40 ± 4	42 ± 4	515 ± 50	1.57 ± 0.13	1.45 ± 0.12
	40	26 ± 3	28 ± 3	452 ± 45	1.71 ± 0.13	1.59 ± 0.13
	50	24 ± 2	26 ± 2	448 ± 40	2.08 ± 0.17	1.78 ± 0.15

## Data Availability

Not applicable.
